# Electronic properties and low lattice thermal conductivity (*κ*_l_) of mono-layer (ML) MoS_2_: FP-LAPW incorporated with spin–orbit coupling (SOC)

**DOI:** 10.1039/d0ra02585b

**Published:** 2020-05-19

**Authors:** D. P. Rai, Tuan V. Vu, Amel Laref, Md. Anwar Hossain, Enamul Haque, Sohail Ahmad, R. Khenata, R. K. Thapa

**Affiliations:** Physical Sciences Research Center (PSRC), Department of Physics, Pachhunga University College Aizawl-796001 India; Division of Computational Physics, Institute for Computational Science, Ton Duc Thang University Ho Chi Minh City Vietnam vuvantuan@tdtu.edu.vn; Faculty of Electrical & Electronics Engineering, Ton Duc Thang University Ho Chi Minh City Vietnam; Department of Physics, College of Science, King Saud University Riyadh Saudi Arabia; Department of Physics, Faculty of Science, Mawlana Bhashani Science and Technology University Santosh Tangail-1902 Bangladesh; Department of Physics, Faculty of Science, King Khalid University Abha Saudi Arabia; Laboratoire de Physique Quantique de la Matière et de la Modélisation Mathématique (LPQ3M), Faculté des Sciences, Université de Mascara Mascara 29000 Algeria; Department of Physics, Mizoram University Aizawl-796004 India

## Abstract

This paper focuses on the electronic and thermoelectric properties of monolayer MoS_2_. Here, we have examined the structure of MoS_2_, in which the hole in the center of the hexagonal cage is considered as a void atom, termed 1H-MoS_2_. Density functional theory (DFT) employing the generalized gradient approximation (GGA) and spin–orbit coupling (SOC) has been used for all calculations. Incorporation of SOC resulted in a significant change in the profile of the band energy, specifically the splitting of the valence band maximum (VBM) into two sub-bands. The “split-off” energy is found to be ∼20.6 meV. The reduction of the band gap with SOC is a prominent feature at the K–K location in the Brillouin zone. The band gap calculated with the GGA is ∼1.75 eV. However, on implementation of SOC, the GGA band gap was reduced to ∼1.68 eV. The frequency-dependent phonon dispersion curve was obtained to analyse the thermodynamical stability. 1H-MoS_2_ is found to be thermodynamically stable with no imaginary frequency. We report a low value of lattice thermal conductivity (*κ*_l_) and low electron effective masses, which are desirable for potential applications in thermoelectric devices.

## Abstract 2

This paper focuses on the electronic and thermoelectric properties of monolayer MoS_2_.

## Graphical abstract

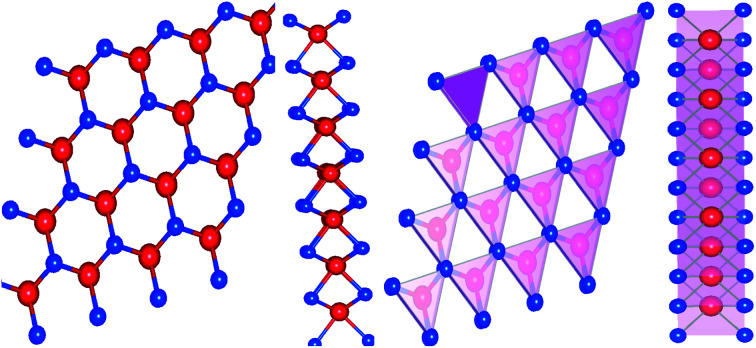

## 1 Introduction

The non-existence of an energy band gap in graphene has seriously hindered its technological applications in digital electronics and other low-power devices.^1^ The technological limitations of graphene are highly challenging and have forced researchers to look for potential 2D materials with finite band gaps.^2,3^ Ultrathin 2D layered materials like single-layer transition metal dichalcogenides (TMDs) (Mo/WX_2_, X = S, Se, Te) with two-fold valley degeneracy are promising due to their appreciable band gap which depends on the thickness and the fact that they exhibit outstanding mechanical properties like those of graphene. TMDs have many industrial applications, such as lubricants,^4^ photo-catalysis,^5^ photo-voltaics^6^ and energy storage.^7^ In particular, MoS_2_ is an important TMD due to its availability and room temperature stability. Furthermore, this material can be synthesized without much effort *via* various experimental techniques, such as chemical vapor deposition,^8,9^ micro-exfoliation^10^ or solvent-based technology.^11,12^ Multi-layer MoS_2_ has a broad commercial application as a dry lubricant which is due to the weak interlayer van der Waals (vdW) interactions between the adjacent layers.^13^ Bulk MoS_2_ is an indirect band gap semiconductor with an energy band gap of ∼1.23 eV, while single layer MoS_2_ exhibits a direct band gap of ∼1.8 eV.^14,15^ The size-dependent tunability of the electronic properties makes MoS_2_ a novel material for nanoscale field-effect transistors and optical sensors.^16–19^ Recently, a hetero-junction layer structure of MoS_2_–HfO_2_ was successfully constructed and implemented in a nanoscale field-effect transistor (NFET).^20^ A WS_2_/MoS_2_ heterojunction was mechanically fabricated and the room temperature photoluminescence spectra were studied by theory and experiments.^21^ Several theoretical studies are in progress, focusing on the physical and chemical properties of 2D MoS_2_ under different applied fields using an *ab initio* approach.^22–25^ Besides the above mentioned functional properties, TMDs have interesting thermoelectric properties which can be utilized in emerging energy harvest applications.^26–29^ Thermoelectric materials have potential technological importance in converting industrial waste heat into electrical energy and *vice versa*.^30–33^ Several studies have reported that TMDs could be potential thermoelectric materials due to their low lattice thermal conductivities and high charge mobilities due to their small effective masses, but the bench mark performance is still in the nascent stage in terms of practical applications.^34–38^ The thermoelectric performance of a solid-state material depends on the dimensionless figure of merit called thermoelectric efficiency (*ZT*), calculated as1
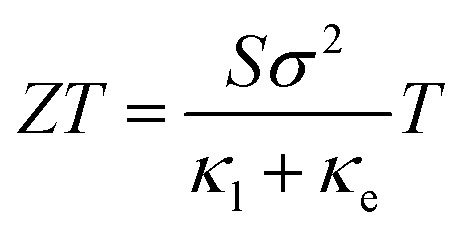
where *S* is the Seebeck coefficient, *σ* is the electrical conductivity, *κ*_e_ is the electronic thermal conductivity and *κ*_l_ is the lattice thermal conductivity. A good thermoelectric material possesses high *S*, high *σ* and low thermal conductivity, *κ* = *κ*_e_ + *κ*_l_. Narrow band gap (0.5–1.8 eV) semiconductors are preferable, and MoS_2_ looks promising in this regard and could have a high *ZT* value. Recently, a high power factor value (*P* = *Sσ*^2^) of ∼8.5 mW m^−1^ K^−1^ has been experimentally reported in few-layer MoS_2_ at room temperature.^39^ On the other hand, the thermoelectric efficiency (*ZT*) of single layer MoS_2_ is very low (0.11 at 500 K).^40^ Huang *et al.*,^41^ using a ballistic model, reported an improved *ZT* value of up to 0.5 (300 K). Other monolayer TMDs like PdS_2_,^38^ MoSe_2_,^42^ WSe_2_,^42^ WS_2_ (ref. 43) and SnSe_2_ (ref. 44) also show significant thermoelectric response with *ZT* values of 0.1 (1200 K), 0.8 (1200 K), 0.90 (1500 K), 1.1 (300 K) and 2.95 (800 K), respectively. However, on structural modification the *ZT* value has been enhanced up to 3.5 at 300 K for armchair nanoribbons (ACNRs), and also bilayer MoSe_2_ shows a maximum room temperature *ZT* value of ∼2.4.^34,40^ Arab *et al.* have also reported high values of *ZT* = 4.0 in 3-ACNRs for n-type MoS_2_ and *ZT* = 3 in 4-ACNRs for p-type MoS_2_ at 500 K.^45^ As we know, *ZT* is inversely related to the thermal conductivity (*κ* = *κ*_e_ + *κ*_l_). For instance, monolayer ZrS_2_, ZrSe_2_ and HfSe_2_ exhibit low *κ*_l_ values of 3.29,^46^ 1.2 and 1.8 W m^−1^ K^−1^,^47^ respectively at 300 K, and the respective *ZT* values are 1.65, 0.87 and 0.95. Even smaller values of *κ*_l_ have been reported for disordered 2D-MoS_2_ and 2D-WSe_2_, *i.e.* 0.05 W m^−1^ K^−1^ and 0.1–1 W m^−1^ K^−1^,^48,49^ respectively. Moreover, the *κ*_l_ value of MoS_2_ nanoribbons was found to be ∼5 W m^−1^ K^−1^ at room temperature using molecular dynamics (MD) theory.^50^ Different values of *κ*_l_ have been reported for 2D-MoS_2_ based on different approaches, *viz.* 1.35 W m^−1^ K^−1^, 23.2 W m^−1^ K^−1^ and 26.2 W m^−1^ K^−1^ obtained from molecular dynamics (MD),^51^ a non-equilibrium Green’s function,^52^ and the Boltzmann semi-classical transport equation (BTE),^36^ respectively. However, some articles have reported high values of *κ*_l_; 83 W m^−1^ K^−1^,^53^ 52 W m^−1^ K^−1^ (ref. 35) and 85–110 W m^−1^ K^−1^ (ref. 54) were observed for the vapour phase of few-layer MoS_2_ and the (001) orientation of a MoS_2_ crystal with basal-plane thermal conductivity as a function of laser spot size. The non-uniformity of thermoelectric responses arises due to the variation in the values of *κ*_l_. Recently, Kaur *et al.*, using the semi-classical Boltzmann transport approach, reported low values of *κ*_l_ of around 8.3 and 5 W m^−1^ K^−1^ for 2D ScP and ScAs, respectively.^55^ A large value for the Seebeck coefficient of −4 × 10^2^ to −1 × 10^5^ μV K^−1^ has also been reported in monolayer MoS_2_.^56^ It seems that the studies of the thermoelectric behaviour of MoS_2_ are still very crude. Therefore, we need a more rigorous and accurate study for the concrete determination of the *κ*_l_ value of MoS_2_. In this paper, an investigation has been carried out on the thermoelectric response of 2D 1H-MoS_2_ using the first principles method incorporating spin–orbit coupling (SOC) along with the semi-classical Boltzmann Transport Equation (BTE) as implemented in BoltzTraP code.^57^

## 2 Computational details

**Fig. 1:**
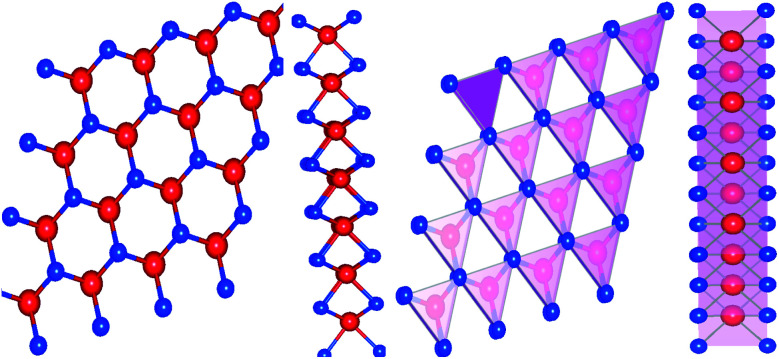
Top view and side view of the 2D structure of 1H-MoS_2_ (Mo – red and S – blue with a polyhedral cage).

The electronic and phonon properties are computed based on Kohn–Sham density functional theory (KS-DFT) using the two computational packages WIEN2K^58^ and QUANTUM ESPRESSO,^59^ respectively. WIEN2K relies on the full-potential linearized augmented plane wave (FP-LAPW) method, whereas QUANTUM ESPRESSO incorporates an ultrasoft pseudopotential. A generalized gradient approximation (GGA) developed by Perdew–Burke–Ernzerhof (PBE)^60^ has been considered for electron exchange–correlation. The valence and semi-core state electrons are treated relativistically considering the spin–orbit coupling (SOC). A dense optimized 10 × 10 × 1 *k*-mesh is adopted for the first Brillouin zone integration in which 286 irreducible *k*-points are used for the energy calculations. The convergence criterion for the complete self-consistency calculation is set as 0.0001 Ry. Bulk MoS_2_ crystallizes in a hexagonal structure with point group 6/*mmm*, space group *P*6_3_/*mmc* and lattice constants *a* = *b* = 3.19 Å, *c*/*a* = 3.86 Å.^61^ A 2D slab was constructed by taking a 10 Å vacuum along the *z*-axis with a void in the center of the hexagonal ring. The 2D slab was optimized by taking the minimum energy until the self-consistent calculation reached below the Hellmann–Feynman force of 0.01 Ry Å^−1^. The optimized lattice constant was found to be *a* = *b* = 3.185 Å and agrees well with the previous finding of 3.183 Å.^62^ The hexagonal crystal structure of monolayer MoS_2_ (both top and side views) is depicted in Fig. 1. To determine the phonon spectrum of MoS_2_, we used a 16 × 16 × 1 *k*-mesh and a 2 × 2 × 1 *q*-mesh. The electron and lattice parts of the thermoelectric parameters were calculated with a 16 × 16 × 1 *k*-mesh by using the first-principles Boltzmann semi-classical transport equation with a single-mode relaxation-time approximation called BoltzTraP^57^ and Phono3py.^63^ BoltzTraP is used to calculate the electron part of the thermoelectric parameters and Phono3py determines the lattice thermal conductivity (*κ*_l_).

## 3 Results and discussion

### 3.1 Electronic properties

**Fig. 2:**
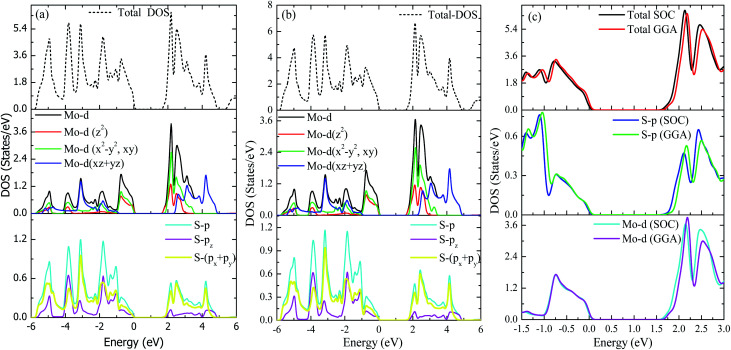
Total and partial DOS of MoS_2_. (a) GGA, (b) GGA–SOC and (c) both GGA and GGA–SOC together.

**Fig. 3:**
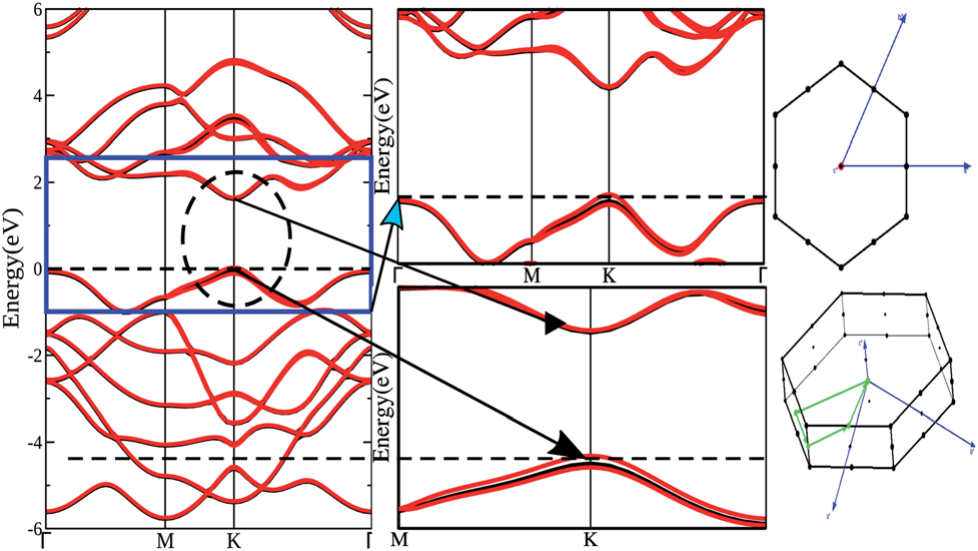
Band structure of 1H-MoS_2_ (GGA: black lines and GGA–SOC: red lines), first Brillouin zone, and primitive cell (blue arrows indicate reciprocal lattice vectors, green lines represent the high symmetry points).

**Fig. 4:**
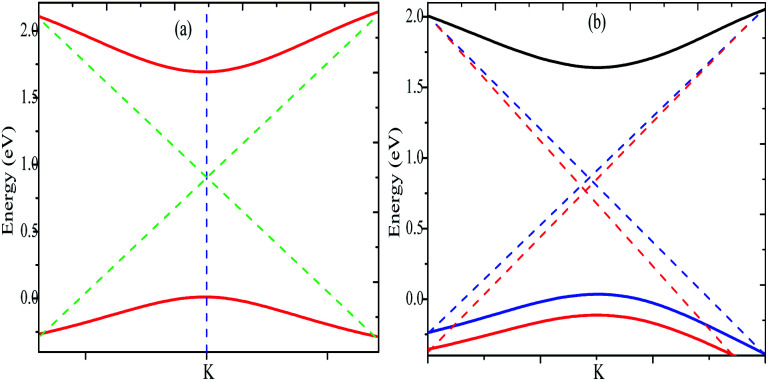
Valence band maxima and conduction band minima calculated with (a) GGA and (b) GGA+SOC.

Top and side views of the 2D structure of MoS_2_ are presented in Fig. 1. The lattice arrangement of MoS_2_ shows clear bonds between the S and Mo atoms, keeping a void or hollow in the central part. Therefore, the top view exactly resembles a 2D graphene-like structure. Six S atoms and one Mo atom form two symmetrical pyramidal polyhedrons, and the Mo atom is at the junction of the two pyramids. The presence of bonding between the S and Mo atoms may be the reason for p–d hybridization which leads to metallic bonding and the absence of van der Waals interactions. The electronic properties are investigated by calculating the density of states (DOS) and band structure of MoS_2_ as shown in Fig. 2 and 3. Based on both (GGA and SOC) calculations, MoS_2_ exhibits a clear band gap at the Fermi level (*E*_F_), showing the existence of semiconducting behaviour. The maxima and minima of the dispersed bands are observed at the high symmetry K point. The probability of electron transitions along the K–K symmetry points indicates that MoS_2_ is a direct band gap semiconductor. The origin of the energy band gap is due to the Mo-d orbital and S-p orbital hybridization, as discussed elsewhere.^14,61,64–67^ For further elucidation of the band structure we have calculated the partial density of states (PDOS) as well. Fig. 2(a and b) display the partial DOS calculated with GGA and GGA–SOC, respectively. A comparison of the total and partial DOS calculated with GGA and SOC is also presented in Fig. 2(c). Fig. 2(a and b) are divided into three layers: top (total DOS), middle (partial DOS of Mo atoms) and bottom (partial DOS of S atoms). The PDOS of the S atoms represents the 3p states. The first relative magnitude of the 3p states of the S atoms in monolayer MoS_2_ (0.31 states per eV) is the same for both GGA and GGA+SOC calculations [Fig. 2(a and b)]. The above results obtained from Fig. 2 and 3 indicate that monolayer MoS_2_ is a direct band gap semiconductor with electron transitions along the K-symmetry points. The major contribution is attributed to Mo-d and S-p states. The valence band (VB) in the range from 0 to −6 eV is an admixture of both Mo-d and S-p states due to the p–d orbital hybridization [Fig. 2(a)]. Whereas, in the conduction band (CB), the energy range from 1.75 to 5 eV is mainly composed of occupied Mo-d states with a small contribution from the S-p states. From Fig. 3 (right side), we can see the significant effect of GGA+SOC on the electronic band structure with the splitting of the valence band maximum (VBM). We have observed that the completely filled Mo-d_*z*^2^_ state lies at the VBM (Fig. 3). Meanwhile on implementation of GGA+SOC, the occupied Mo-d_*z*^2^_ state is pushed towards lower energy while the Mo-d_*x*^2^−*y*^2^,*xy*_ band is ∼0.026 eV higher in energy on approaching the *E*_F_ [Fig. 3 (middle bottom line marked by an arrow head) and Fig. 4]. This “split-off” energy is almost half of the experimentally measured value of 0.042 eV.^68^ Also, the unoccupied Mo-d_*z*^2^_ state at the CBM drops down by a small amount of energy as compared to the GGA band, as shown in Fig. 2(b). Thus, GGA+SOC reduces the GGA band gap from ∼1.75 eV to ∼1.68 eV. The presence of two degenerate bands at the VBM along the K-symmetry point on the application of SOC is probably due to the Mo-d_*x*^2^−*y*^2^,*xy*_ and Mo-d_*z*^2^_ states.

### 3.2 Thermoelectric properties

**Fig. 5:**
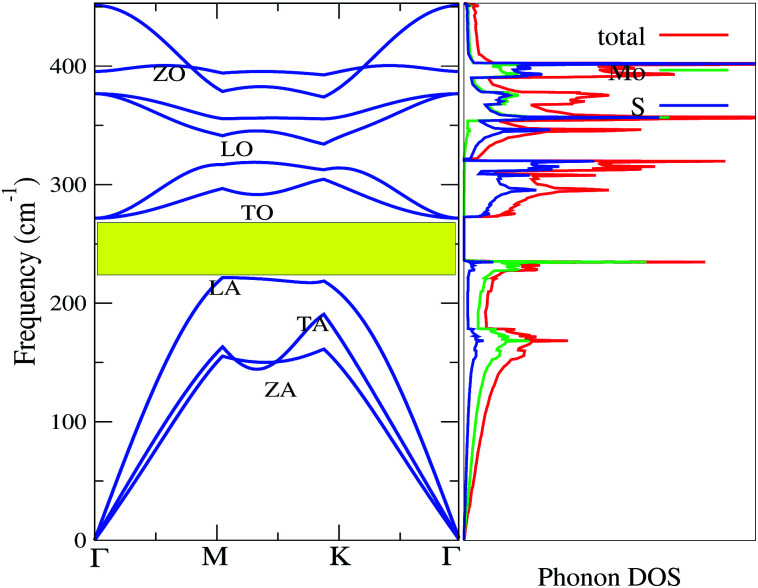
Phonon bands and phonon DOS of 2D monolayer MoS_2_.

**Fig. 6:**
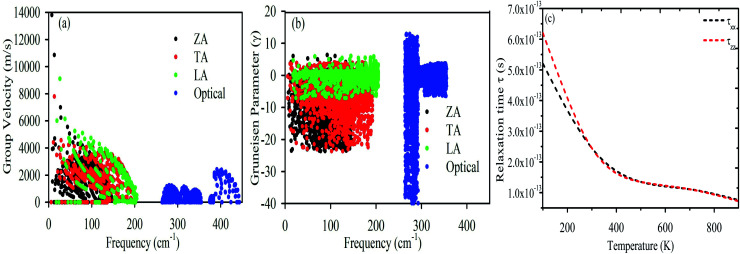
(a) Group velocity, (b) Grüneisen parameter and (c) phonon relaxation time (*τ*_p_).

To confirm the thermodynamical stability for practical synthesis, we have calculated the frequency-dependent phonon dispersion using density functional perturbation theory (DFPT)^63,69,70^ as shown in Fig. 5. The presence of phonon modes in the positive frequency range indicates that monolayer MoS_2_ is dynamically stable for laboratory synthesis. The three atoms in the primitive cell give nine distinctive vibrational modes along the Γ-point. The nine phonon branches are mixtures of three acoustic (lower frequency) and six optical (higher frequency) branches. As shown in Fig. 5, the acoustic modes are identified as the transverse mode (TA), longitudinal mode (LA), and out-of-plane mode (ZA) whereas the optical branches are composed of two transverse modes (TO) at the bottom, two longitudinal modes (LO) in the middle and two out-of-plane modes (ZO) at the top. The optical mode (TO) and acoustic mode (LA) are well separated by ∼52 cm^−1^. The finite frequency band gap as a result of LA–TO splitting may be attributed to the large difference in the atomic masses (Mo = 95.96 amu and S = 32.06 amu). The optical branches along the Γ symmetry points are at ∼275 cm^−1^, ∼370 cm^−1^, ∼395 cm^−1^ and ∼460 cm^−1^, consistent with the previous results.^37,62^Fig. 6 demonstrates the phonon transport related parameters: (a) group velocity at 300 K, (b) Grüneisen parameter at 300 K and (c) relaxation time (*τ*). The three acoustic modes have the highest group velocity and Grüneisen parameter at a particular frequency and *q*-point. This indicates high phonon–phonon scattering rates in which most of the heat is transported by the transverse acoustic mode (TA) and longitudinal acoustic mode (LA).^71,72^ Therefore, the lattice thermal conductivity can be further reduced by doping with the dissipation of heat that may be introduced by the acoustic–optical phonon scattering channels.^73^ The group velocity of the ZA mode can reach ∼14 km s^−1^ at a particular phonon frequency. However, the observation of low group velocity at higher frequencies for the optical modes indicates a small but significant contribution to the thermal conductivity. Anharmonicity results in enhanced phonon–phonon scattering, which reduces *κ*_l_ without affecting the electronic properties.^74^ The Grüneisen parameter measures the strength of the anharmonicity. Therefore, the larger the Grüneisen parameter, the stronger the phonon scattering. The high value of the Grüneisen parameter suggests high anharmonicity in 2D-MoS_2_ and intense phonon scattering. The short phonon relaxation time of 2D-MoS_2_ also indicates intense phonon scattering.

The description of electron transport in a solid-state material is directly related to the electronic energy bands which give rise to a thermoelectric response measured in terms of a dimensionless figure of merit *ZT* as already presented in eqn (1). As reported earlier, monolayered MoS_2_ is a semiconductor with a direct band gap value of ∼1.8 eV.^14,64,67^ In semiconductors, the effective mass (m*) plays a vital role in deriving the quantitative transport characteristics. The effective mass of a charge carrier can be obtained from the parabolic band near *E*_F_ based on the energy-independent scattering approximation^79,80^ given by eqn (2).2
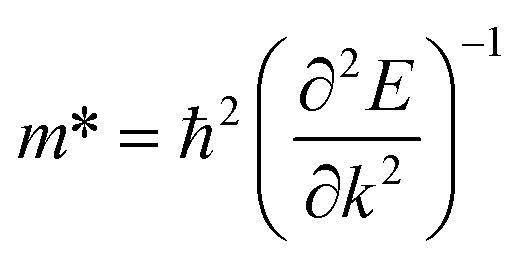


The effect of SOC leads to splitting of the band near *E*_F_ close to the VBM which results in two effective masses for holes [*cf.*Fig. 3 and 4]. A lower value of effective mass leads to a higher value for the charge mobility. On the other hand, the electrical conductivity (*σ*) is directly related to the charge carrier mobility *via*eqn (3)

3 *σ* = *neμ*

where *n* is the concentration of charge carriers and *e* is the electron charge. The calculated effective masses for electrons 
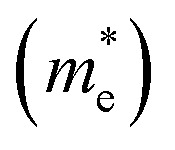
 and holes 
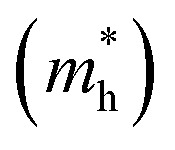
 are presented in Table 1. Our calculated values of the effective masses are 
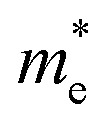
 = 0.391 (GGA), 
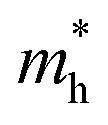
 = 0.552 (GGA), 
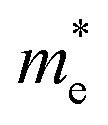
 = 0.315 (SOC) and 
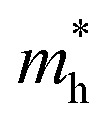
 = 0.467 (SOC); these values are in good agreement with the earlier results estimated with the GGA,^75^ DFT-HSE^78^ and tight binding (TB)^78^ approaches. The lattice part of the thermal conductivity *κ*_l_ has been determined by using the Phono3py code^63^ which iteratively solves the BTE from eqn (4):4
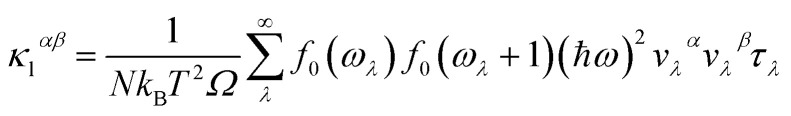
where *Ω* is the primitive cell volume, *α* and *β* are the Cartesian components, *k*_B_ is the Boltzmann constant, *ω*_*λ*_ and *v*_*λ*_ are the angular frequency and group velocity, *τ*_*λ*_ denotes the phonon relaxation time and *f*_0_ is the Bose–Einstein distribution function near *E*_F_.

**Table 1:** Calculated energy band gap (*E*_g_) in eV and effective masses (m*) of electrons 
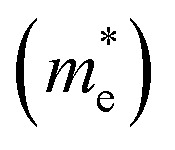
 and holes 
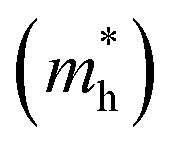
 in terms of electron mass (*m*_e_)

Functional	Energy band gap				m*		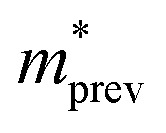
	CBM	VBM	*E*_g_	Prev. *E*_g_	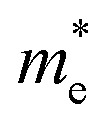	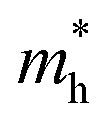	
GGA	−0.02	1.73	1.75	1.77 (ref. 75)	0.391	0.552	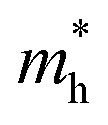 = 0.60, 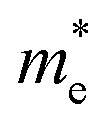 = 0.46,^75^ 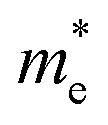 = 0.54 (ref. 77)
				1.77 (ref. 76)			
				1.78 (ref. 77)			
				1.786 (ref. 78)			
GGA–SOC	0.00	1.68	1.68		0.315	0.467	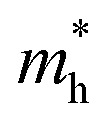 = 0.485/0.463, 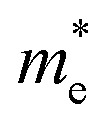 = 0.407/0.430 (ref. 78)
							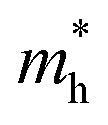 = 0.49, 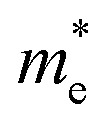 = 0.44 (ref. 76)

The electron parts of the thermoelectric parameters are given by eqn (5)–(9) as presented in the BoltzTraP user manual.^57^

Here *σ*_*α*,*β*_ are the electrical conductivity tensors,5
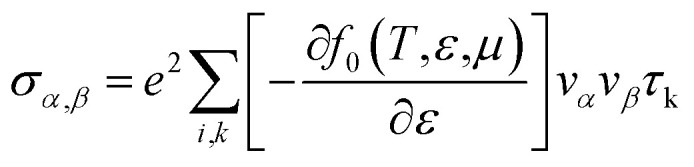
where *α* and *β* refer to the tensor indices, *v*_*α*_ and *v*_*β*_ are the group velocities, *e* is the charge of the electron and *τ*_k_ is the electron relaxation time. The contributions of the electrons are mostly found near the Fermi energy (*E*_F_), termed as the chemical potential (*μ*) (*μ* − *k*_B_*T* < *ε* < *μ* + *k*_B_*T*) where *k*_B_ is the Boltzmann constant. The kernel of the transport distribution is given by:6
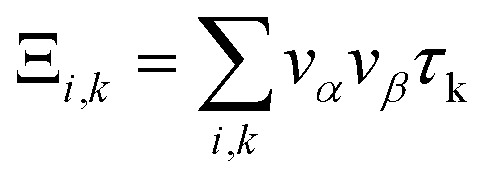


The electron relaxation time (*τ*) dependent electrical conductivity (*σ*/*τ*), thermal conductivity (*κ*/*τ*) and Seebeck coefficient can be written as7
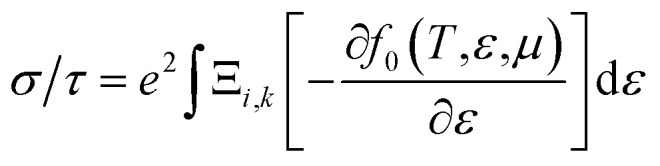
8
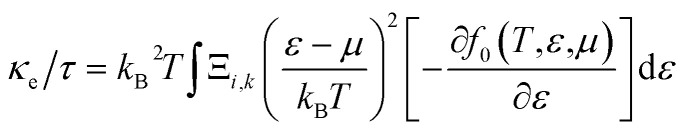
9
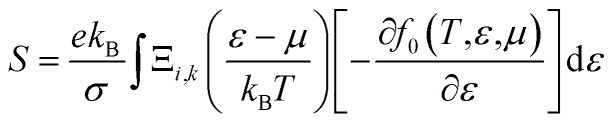
where *f*_0_ is a Fermi–Dirac distribution function.

**Fig. 7:**
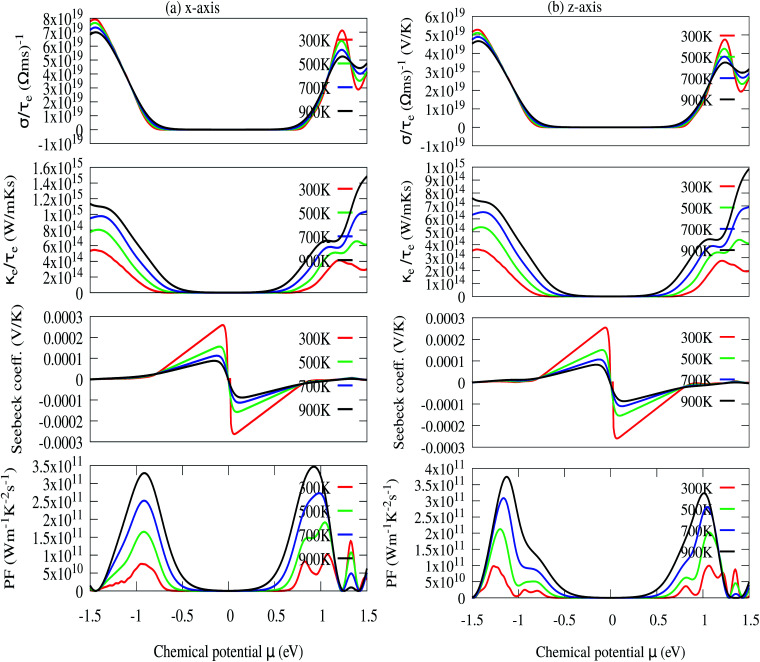
(a) In-plane *x*-axis: electron relaxation time (*τ*_e_) dependent electrical conductivity (*σ*/*τ*_e_), electron thermal conductivity (*κ*_e_/*τ*_e_), Seebeck coefficient (*S*) and power factor (PF), (b) out-of-plane *z*-axis: electron relaxation time (*τ*_e_) dependent electrical conductivity (*σ*/*τ*_e_), electron thermal conductivity (*κ*_e_/*τ*_e_), Seebeck coefficient (*S*) and power factor (PF).

**Fig. 8:**
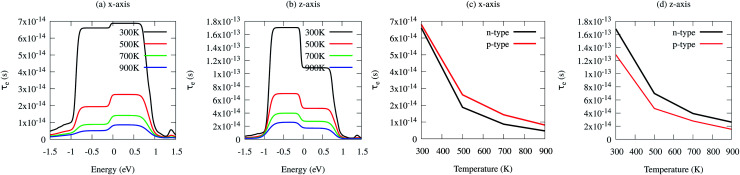
(a) Electron relaxation time (*τ*_e_) as a function of chemical potential along the *x*-axis, (b) electron relaxation time (*τ*_e_) as a function of chemical potential along the *z*-axis, (c) electron relaxation time (*τ*_e_) as a function of temperature along the *x*-axis and (d) electron relaxation time (*τ*_e_) as a function of temperature along the *z*-axis.

The relaxation time (*τ*_e_) dependent electrical conductivity (*σ*), Seebeck coefficient (*S*), electron thermal conductivity (*κ*_e_) and power factor (PF) as a function of chemical potential (eV) along the *x*- and *z*-axes are shown in Fig. 7(a and b), respectively. The electron parts of the thermoelectric parameters are strongly dependent on the electron relaxation time (*τ*_e_). The thermoelectric efficiency (*ZT*) value cannot be estimated unless and until *τ*_e_ is decoupled. Therefore, we have performed the electron transport calculation by using the modified BoltzTraP code based on the electron carrier relaxation time. The expression for electron relaxation time is given by eqn (10)^82,83^10
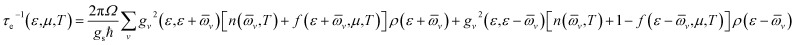
where *Ω* refers to the volume of the primitive cell, *ℏ* is Planck’s constant, *v* is the phonon mode index, *ω̄*_*v*_ is the averaged phonon mode energy, *g*_*v*_^2^ is the averaged electron–phonon matrix, *n*(*ω̄*_*v*_,*T*) is the Bose–Einstein distribution function, *f*(*ε* + *ω̄*_*v*_,*μ*,*T*) refers to the Fermi–Dirac distribution function, *g*_s_ = 2 is the spin degeneracy, *ε* is the electron energy, and *ρ* is the density of states per unit energy and unit volume (*V*). The electron relaxation times along both the *x*- and *z*-axes as a function of chemical potential and absolute temperature are presented in Fig. 8(a–d). The electron relaxation times along the *z*-axis are longer than those along the *x*-axis for both n-type and p-type carriers. However, the electron relaxation times of n-type and p-type carriers along the same axis are almost the same. The electrons in the conduction band which lies close to the Fermi energy shows longer relaxation times as compared to the holes in the valence bands along the *x*-axis. This is in contrast to the carriers along the *z*-axis. For both carriers, *τ*_e_ decreases with an increase in absolute temperature. The sharp decrease in *τ*_e_ near the band edges can be visualised as eqn (11)^82^

11 *τ*_e_^−1^ = ∼*g*^2^(*ε*)*ρ*(*ε*)

Eqn (11) established an inverse relation between *τ*_e_ and the carrier density of states per unit energy and volume (*ρ*), while the electron–phonon matrix elements (*g*) depend weakly on the energy. The *τ*_e_ value increases from ∼1 × 10^−14^ s to ∼7 × 10^−14^ s along the *x*-axis and from ∼2.0 × 10^−14^ s to ∼1.8 × 10^−13^ s along the *z*-axis as the temperature decreases from 900 K to 300 K [see Fig. 8(a–d)].

**Fig. 9:**
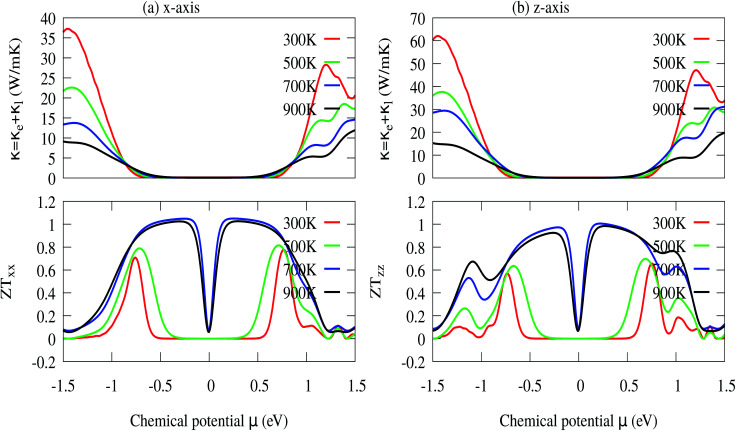
(a) In-plane *x*-axis: total thermal conductivity (*κ* = *κ*_e_ + *κ*_l_) and *ZT*_*xx*_, and (b) out-of-plane *z*-axis: total thermal conductivity (*κ* = *κ*_e_ + *κ*_l_) and *ZT*_*zz*_.

The optimized values of the thermoelectric parameters obtained at 300 K are presented in Table 2. The high value of *S* and low *κ*_l_ are indicative that the monolayer MoS_2_ system can be a prospective candidature for thermoelectric applications. However, the presence of a wide band gap in monolayer MoS_2_ may result in a low value of electrical conductivity (*σ*). The use of SOC has a significant effect on the band energy of MoS_2_ due to the sizeable spatial expansion of the Mo-3d-orbital which may lead to desirable physical and chemical properties. The spin–orbit interaction is applied along the easy spin quantization axis [001] direction. We found that the large GGA band gap of the monolayer MoS_2_ semiconductor was reduced to ∼1.68 eV which may enhance the power factor. Hence, we have calculated all the thermoelectric properties using GGA–SOC. The calculated *σ* for both n-type and p-type carriers along the *x*-axis are found to decrease from ∼5.0 × 10^5^ to ∼5 × 10^4^ Ω^−1^ m^−1^ with an increase in absolute temperature from 300 to 900 K. Similarly, along the *z*-axis, *σ* varies from ∼1.45 × 10^6^ to ∼1.5 × 10^5^ Ω^−1^ m^−1^ in the same temperature range. At room temperature the total thermal conductivities are found to be *κ*_*xx*_ = ∼36.23 W m^−1^ K^−1^ and *κ*_*xx*_ = ∼30.18 W m^−1^ K^−1^ for n-type and p-type carriers, respectively. On the other hand, the calculated room temperature values of the total thermal conductivities along the *z*-axis are almost two times higher, *κ*_*zz*_ = ∼60.00 W m^−1^ K^−1^ and *κ*_*zz*_ = ∼50.84 W m^−1^ K^−1^ for n-type and p-type carriers, respectively. Our results for total thermal conductivity measured along the *x*-axis are consistent with the results obtained from molecular dynamics (23.2 W m^−1^ K^−1^) and from a non-equilibrium Green’s function (26.2 W m^−1^ K^−1^).^52^ Moreover, the results along the *z*-axis agree well with the values of 52 W m^−1^ K^−1^ (ref. 35) and 85–110 W m^−1^ K^−1^ (ref. 54) measured for the vapour phase of few-layer MoS_2_ and the (001) orientation of a MoS_2_ crystal with basal plane thermal conductivity as a function of laser spot size, respectively. We observed a sharp decrease in *κ* as the temperature increased from 300 K to 900 K [Fig. 9(a and b)]. A similar trend has also been reported for WS_2_, in which *κ*_l_ decreases from 150 to 100 W m^−1^ K^−1^within the 200–500 K temperature range. Also, in WSe_2_, *κ*_l_ decreases from 50 to 30 W m^−1^ K^−1^ in the same temperature range.^81^ The sharp rise in DOS near the Fermi energy gives rise to the high value of *S*. The maximum values of *S* are found to be ∼1.19 × 10^−4^ V K^−1^ and ∼1.41 × 10^−4^ V K^−1^ for the n-type and the p-type carriers along the *x*-axis, respectively. Fig. 9(a and b) display the total thermal conductivity (*κ* = *κ*_l_ + *κ*_e_) and figure of merit (*ZT*) as a function of chemical potential along the *x*- and *z*-axes, respectively. For both the *x*- and *z*-axes, the *ZT* value due to hole carriers (p-type) surpasses the n-type *ZT* value. The *ZT* values are found to be 0.60–0.76 at room temperature which seems to be too small for practical applications [Table 2]. Interestingly, the *ZT* increases up to ∼1.00 at 1000 K for p-type carriers (taken from the peak value) [see Fig. 9(a and b)]. The linear behaviour of *ZT* as a function of temperature signifies the potential of monolayer MoS_2_ as a high temperature thermoelectric material.

**Table 2:** Calculated *σ* (Ω^−1^ m^−1^), *S* (V K^−1^), *τ*_e_ (s), *κ* = *κ*_e_ + *κ*_l_ (W m^−1^ K^−1^) and thermoelectric efficiency (*ZT*) at 300 K (all calculations are performed with GGA–SOC)

Carriers	*σ*	*S*	*τ*_e_	*κ* = *κ*_e_ + *κ*_l_	*ZT*
**Along *xx***					
n-type	5.13 × 10^5^	−0.119 × 10^−3^	6.807 × 10^−14^	36.238	0.75
p-type	3.26 × 10^5^	0.141 × 10^−3^	6.602 × 10^−14^	30.180	0.76

**Along *zz***					
n-type	6.192 × 10^5^	−1.12 × 10^−4^	1.648 × 10^−13^	60.00	0.60
p-type	14.07 × 10^5^	1.25 × 10^−4^	1.291 × 10^−13^	50.84	0.62

## 4 Conclusion

In this investigation, we have studied the electronic and thermoelectric properties of monolayer MoS_2_ using the GGA and GGA–SOC approaches. The calculation of the electronic structure shows that monolayer MoS_2_ is a direct band gap semiconductor, as the CBM and VBM lie at the same K-symmetry point. The calculated band gap is found to be 1.75 eV, in good agreement with the previous experimental and theoretical results. Monolayer MoS_2_ exhibits high *S* and low *κ* values, which are crucial for thermoelectric applications. However, with GGA the electrical conductivity (*σ*) is observed to be suppressed which eventually limits the thermoelectric power factor due to the presence of a wide band gap. We have found that SOC has a significant effect on the band energy of monolayer MoS_2_ due to the presence of the larger 4d orbital. A reduction of the direct band gap has been observed on application of spin–orbit coupling along the spin quantization 001 direction. The reduced band gap is expected to enhance *σ* at room temperature. Our calculated thermoelectric parameters are consistent with the available data. The reduced lattice thermal conductivity at elevated temperatures is another interesting feature. As a result, the *ZT* value approaches the benchmark value of ∼1.0 at a temperature of ∼1000 K.

## Conflicts of interest

All authors declare that there are no conflicts of interest.

## Supplementary Material
